# Comparison of binocular game and patching in treating mild to moderate anisometropic amblyopia: a study protocol for a randomized controlled trial

**DOI:** 10.1186/s13063-021-05735-2

**Published:** 2021-10-30

**Authors:** Mohammad Etezad Razavi, Marzieh Najjaran, Jaber Mohseni, Shokoufeh Aalaei

**Affiliations:** 1 Eye Research Center, Mashhad University of Medical Sciences, Mashhad, Iran; 2 Department of Optometry, School of Paramedical Sciences, Mashhad University of Medical Sciences, Mashhad, Iran; 3 Department of Medical Informatics, Faculty of Medicine, Mashhad University of Medical Sciences, Mashhad, Iran

**Keywords:** Amblyopia, Patch therapy, Serious game, Stereopsis, Binocularity, Mobile health

## Abstract

**Background:**

Amblyopia, as a neurodevelopmental preventable visual disorder, affects approximately 1.1 % in Asia. A binocular approach to treating amblyopia has been recently proposed. Whether the binocular playing game treatment is comparable to patching treatment needs further randomized clinical trials. To address this, the present research, designs, develops, and evaluates a new binocular game to treat amblyopia.

**Methods:**

This study has been designed as a non-inferiority, randomized, two parallel-group, controlled trial. Forty-four patients between 4 and 12 years diagnosed with amblyopia will be randomly assigned to the control and intervention groups. In the intervention group, amblyopia treatment is provided with red-green anaglyphic glasses and a red filter placed in front of the amblyopic eye, along with a game to be played for 30 min twice a day. Those in the control group will receive patch therapy according to amblyopia treatment study protocol. The primary outcome is to change visual acuity in the amblyopic eye from the baseline to 3 months after randomization.

**Ethics and dissemination:**

The Ethics Committee of Mashhad University of Medical sciences’ approval date was February 28, 2018, with a reference code of IR.MUMS.fm.REC.1396.783. Thus far, the recruitment of participants has not been completed and is scheduled to end in September 2021. The results will be disseminated in a peer-reviewed journal.

**Trial registration:**

Iranian Registry of Clinical Trials IRCT20180217038768N1. Registered on 22 April 2019.

**Supplementary Information:**

The online version contains supplementary material available at 10.1186/s13063-021-05735-2.

## Introduction

Amblyopia, as a neurodevelopmental preventable visual disorder, affects approximately 1.1% in Asia [1]. It follows from inadequate stimulation of the visual system during the critical period of visual development. Amblyopia is mainly associated with visual acuity (VA) reduction and binocular dysfunction. The most common risk factor for unilateral amblyopia is anisometropia [2, 3].

Refractive correction is the first step of amblyopia treatment regardless of the cause of amblyopia (anisometropia, strabismus, or both) [4, 5]. Patching the healthy eye has a long history and is the current standard treatment with at least one to two line improvements in VA in the amblyopic eyes [6–8]; however, it is faced with several limitations. Abnormal binocularity [9–11], recurrence even after a successful treatment in at least 25% of amblyopic children [12], poor compliance and negative outcomes including distress [13], and low self-perception of social acceptance [12] hinder the success of patching treatment.

Penalizing the fellow eye either with atropine or optically as the alternative options to occlusion therapy along with their effective outcomes needs careful monitoring [14, 15]. Both patching and penalizing methods are monocular treatment approaches, in which by depriving the fellow eye of vision, the use of the amblyopic eye is promoted.

More recently, a novel approach based on the binocular origin of amblyopia has received considerable research interest due to possibly better visual outcomes in treating amblyopia [16, 17].

The relevant hypothesis for the binocular treatment of amblyopia is anti-suppression therapy, according to which the cortical input is suppressed in the amblyopic eye by inhibitory signals from the fellow eye. Thus, through minimizing suppression, the brain learns to see through the amblyopic eye [10]. In this context, the dichoptic presentation stimulus is applied to alleviate suppression in amblyopia.

In dichoptic training, each eye receives different images separately, and to complete a task, both eyes are forced to work together. The fixing eye versus the amblyopic receives the stimuli with the lower contrast. If the task is successfully completed, the contrast in the non-amblyopic eye is slowly increased until that in both eyes is equal.

The association between binocular dysfunction and deficits related to amblyopia has been clarified by Birch et al. [11]. In addition, in children between 3 and 7 years of age, Birch et al. found approximately 1 line improvement of visual acuity in amblyopic eyes with binocular iPad treatment [18]. In another study by Kelly et al. using an adventure binocular game for 1 h a day, they found an improvement of 1.7 lines of VA in 4 weeks [19].

Besides the amblyopic-eye VA improvement with binocular treatment, enhancing binocular functions has been proposed in several studies [5, 20, 21]. Weber et al. reported the improvement of fine motor skills 5 weeks after the binocular treatment in amblyopia [20].

However, despite the promising results of binocular treatment in children with amblyopia [18, 19, 22, 23], randomized clinical trials showed inconsistent results in the amblyopic eye visual acuity improvement [24–26].

Some other research by the Pediatric Eye Disease Investigator Group (PEDIG) did not reveal the priority of binocular game treatment over patching not only to children but also to teenagers with amblyopia [27, 28]. Similarly, Jing Yao et al. showed that although a 40-min daily game played for 3 months could improve VA for 0.18 logMAR, binocular game treatment alone was not more effective than patching in treating children with anisometropic amblyopia. The new computer game applied in the study was based on a push-pull method [26]. In this model, stimulating the amblyopic eye while inhibiting the strong eye led to the re-balancing of intraocular interactions [26, 29].

There is also a considerable need for further robust clinical trials to show the possible effectiveness of binocular treatment. The existing differences among several factors in different studies, including the severity and type of amblyopia, type of binocular game, dose of treatment prescribed, and prior amblyopic treatment, make cross-comparison difficult.

More appealing games and more frequent supervision are suggested in studies exploring binocular amblyopia treatment [19, 26, 30]. Furthermore, by increasing the popularity of video games, they can be used for healthcare purposes with a higher interest in research [31]. With this regard, poor compliance as an issue accounting for patching treatment failure could benefit from such an engagement strategy for amblyopia treatment [17, 18, 22].

Therefore, the present study aims to design, develop, and evaluate a new binocular game using dichoptic images to investigate whether binocular game treatment makes any difference in the visual acuity of target patients.

We hypothesized that game with specific, well-defined characteristics designed in a structured way with the participation of a multi-disciplinary team would improve amblyopic-eye visual acuity in patients who received game compared to patients who only received patch therapy. So, a non-inferiority, randomized, two parallel-group, controlled trial is employed to investigate whether the binocular game makes any difference in visual acuity outcome in target patients.

## Methods

### Study design and setting

This manuscript was written in accordance with the SPIRIT (Standard Protocol Items: Recommendations for Interventional Trials) and CONSORT (Consolidated Standards Of Reporting Trials) 2010 guidelines [32, 33] (Additional file 1).

A non-inferiority, randomized, two parallel-group, controlled trial is employed to investigate whether the binocular game makes any difference in visual acuity in target patients.

The study is conducted in Khatam Alanbia Eye Hospital in Mashhad, Khorasan Razavi province, Northeastern Iran. Khatam Alanbia is the only specialized public hospital affiliated with universities providing ophthalmology services in northeastern Iran. The center is affiliated with Mashhad University of Medical Sciences (MUMS).

### Participants

The target population consists of untreated patients afflicted with mild to moderate anisometropic amblyopia, who refer to the center and meet the inclusion criteria. Parents or the caretakers of child participants will need to provide written informed consent for participation. Similarly, the child participants will give oral informed consent. An assistant researcher will collaborate to obtain the consent. It will be ensured that they can withdraw from the study any time they want with no effect on their subsequent care. Informed consent has already been evaluated by the Ethics Committee of MUMS (Ethical code: IR.MUMS.fm.REC.1396.783). The eligibility criteria are assessed by one of the investigators (JM) as below.

#### Inclusion criteria

- Children aged 4 to 12 years with anisometropic amblyopia (amblyopia in the presence of a spherical equivalent ≥ 0.50 diopter between two eyes or difference in stigmatism in any meridian ≥ 1.50 diopter) with an interocular difference of at least two lines
- Children not previously treated for amblyopia except for spectacle correction with stable best corrected visual acuity (BCVA)
- Children with mild to moderate anisometropic amblyopia (BCVA ≥ 0.2)
- Children whose parents or caretakers consented to enter the study

#### Exclusion criteria

- Patients with amblyopia with other causes (non-refractive)
- Patients with ocular or systemic diseases or any previous intraocular surgery
- Patients with a motor neurological disorder and brain lesions but unable to play the game
- Patients unable or unwilling to provide informed consent or not accessible by the end of the study

### Objectives

The primary purpose of the present trial is to investigate whether a 3-month binocular game treatment is comparable to part-time patching in improving amblyopic-eye visual acuity. We hypothesized a 15% change in visual acuity after binocular game treatment. The trial will also assess any change to stereoacuity and suppression; furthermore, the compliance of the binocular game will be addressed.

### Treatment arms

**Fig. 1:**
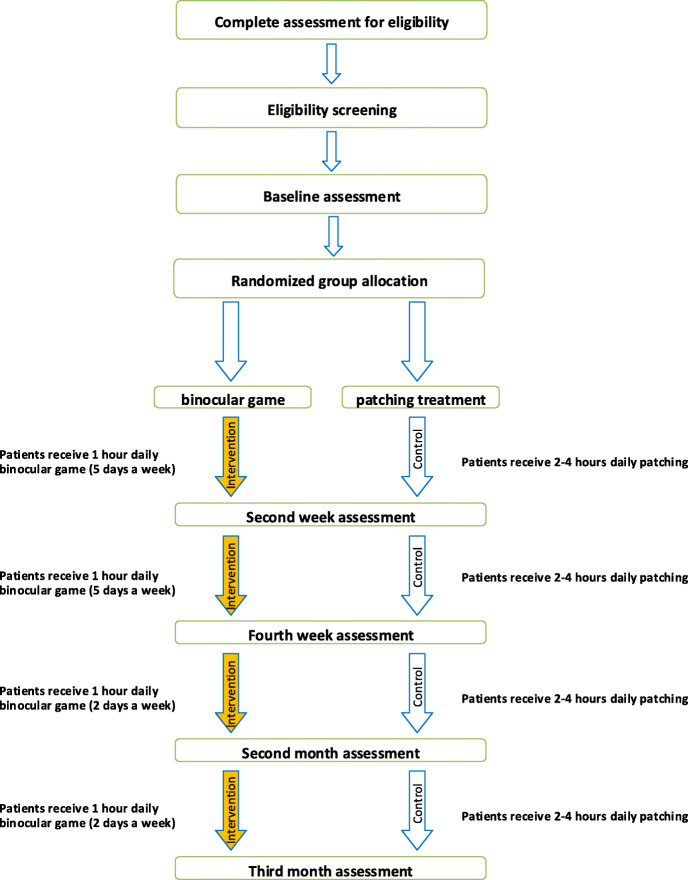
Schematic of the study procedure

Before the randomization, participants with no previous history of using spectacles will have to wear their appropriate optical correction for 16 weeks full-time. After that time, if the visual acuity of the amblyopic eye is stable or a change of 0.1 logMAR or less is observed for another four weeks, and they meet all inclusion criteria, written informed consent will be obtained and randomization will follow the baseline examination. For participants with a history of wearing correction for more than 3 months meeting all eligible criteria, the randomization will follow immediately. Amblyopic children will receive active dichoptic binocular game or patching treatment for 3 months (Fig. 1).

#### Intervention group

In the intervention group, amblyopia treatment is done with red-green anaglyphic 3D glasses with a red filter placed in front of the amblyopic eye and a game for 30 min twice a day (totally 1 h a day), 5 days a week for 4 weeks. Then, this will continue for 2 days a week for 8 weeks (36 h of total treatment).

#### Control group

In the control group, according to the amblyopia treatment study protocol, participants will be required to patch their non-amblyopic eye for 2–4 h a day and last for 3 months. They will receive three times in-person clinical assessments between baseline and final assessment as well as the intervention group.

### Intervention

#### Need analysis

To design the game to treat amblyopic patients, the first step was to extensively review the related literature on the games already used in terms of the effectiveness, appropriateness to the target group, specific features of the game, and probable discussions in design. Moreover, the existing games on amblyopia in AppStore were analyzed. Then, in several meetings with eye specialists (*n* = 3), the scientific aspects of the topic and the characteristics of target patients in the game and their compliance to traditional treatments and the necessity of offering an alternative or complementary therapy were analyzed. Consequently, according to the present findings and experts’ comments and patients’ cultural and local tendencies, the scenarios and main features of the game were specified for the design.

#### Design process

**Fig. 2:**
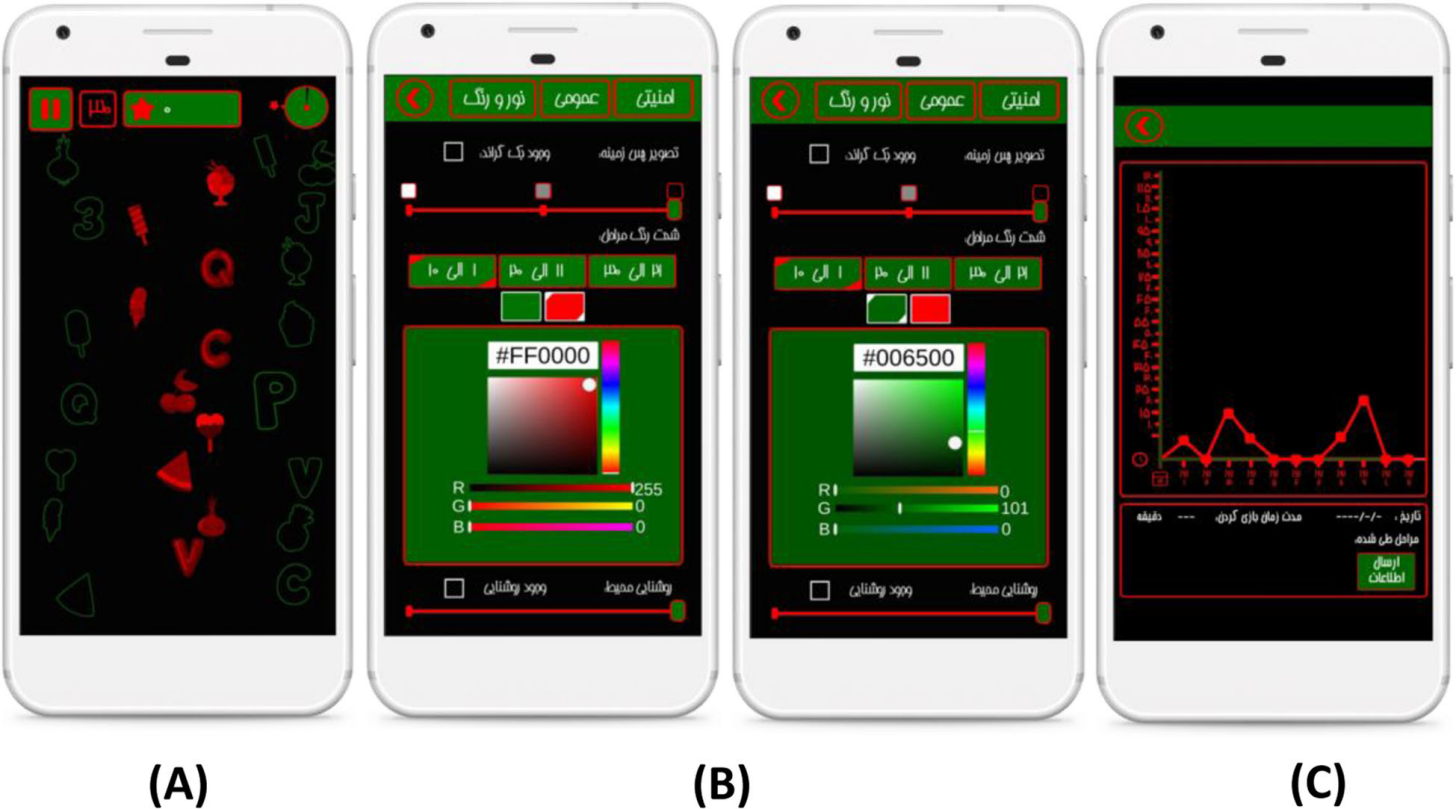
Screenshot of **A** a sample page of binocular Pivot game, **B** setting page, and **C** statistics page

To design the technical features of the game and apply the maximum capabilities of the game production domain, a team of experts was consulted to design and develop the game. The original idea of the game was discussed in meetings shared between the clinical and technical teams. The ambiguities were solved and the technical aspects were discussed. Eventually, considering the fact that the amblyopic eye should be exposed to more complex and dynamic images and static images for stronger eyes, a dichoptic game (Pivot) is designed to enhance binocularity. The images in this game are designed to be used with red-green anaglyphic 3D glasses to filter out green and red images for amblyopic and fellow eye, respectively. Red dynamic figures were seen only by the amblyopic eye and green static figures only by the fellow eye, without attenuating the contrast of the fellow eye (Fig. 2A).

During the design procedure, a formative evaluation was used and the feedback received from the experts helped remove the existing defects. The major changes made to the game were: adding to the number of constituent levels, adding to the variety of graphical forms applied in different levels and adding specific options to settings and statistics.

#### Game features

The game consists of 30 main levels initially locked except the first level and would be unlocked by playing and winning each level. The user must be able to place the red images in the green frames to move on to the next level and obtain bonus points to open the mini-games. Mini-games were in line with the original game system, and to add more attraction, each level had its properties and challenges.

Overall, there are 200 levels in the game, which can help the user stay in and add to the playing time and thus probably help to improve amblyopia via the binocular game.

Based on game design principles, to add to the game's attraction and motivate users, various adaptations have been made according to user’s speed, focus on the least frequent error, duration of daily play and community in gaming, and so on.

An essential part of the Pivot game is the settings and statistics pages. In the settings, you can set a background image and background color, select a color code for static and dynamic figures according to the hexadecimal color coding system, and adjust the color intensity of each ten levels, the ambient brightness, and the game speed (Fig. 2B).

By default, the speed of the game at each level is increased to stimulate the amblyopic eye. Moreover, adjusting the speed enables the physician to make changes to speed according to the patient player’s age, skills, and comments.

In the statistics, there is a graphical presentation of the time, date, and duration of the game, which allows the physician to obtain the required information about the user’s activity at a glance. This feature enables the physician to look for possible reasons for not playing the game and, consequently, recommend alternative treatments if necessary (Fig. 2C).

Moreover, there is an option for sending information in more detail, including demographic data recorded at the beginning of the game, game date, game duration, game level, the frequency of winning and losing in each level, and so on in the form of Excel file online provided to the server.

#### Pilot implementation of Pivot

In the first step, to evaluate the game in terms of the scenarios and appropriateness of goals, the game was evaluated by eye specialists. After applying the expert comments and getting their approval, in the second step, to get patients’ comments in the target group, the game was provided to a limited sample of patients meeting the inclusion criteria (*n* = 5). After a week, they were questioned about their experience. The focus was on their satisfaction with the game and their willingness to use the game. The feedbacks acquired showed their satisfaction with the game and willingness to continue. Moreover, their suggestions to improve the quality of the game were included.

### Outcome measures

#### Primary outcome

Primary and secondary outcomes are measured at baseline and 2 weeks, 1 month, 2 months, and 3 months after the baseline.

The primary outcome is the change to visual acuity in the amblyopic eye from baseline to three months after randomization.

#### Secondary outcomes

- Change to visual acuity in the amblyopic eye after 2 weeks and 1, 2, and 3 months of randomization
- Change to two binocular outcomes, including 1) stereoacuity (using Randot Stereotest) and 2) interocular suppression (Worth Four-dot Test at 6 m and 33 cm)
- Compliance with treatment as at least 25% of the recommended time to play the game during the study (9 h) [5]

### Data collection tools

Uncorrected and best corrected visual acuity (UCVA and BCVA) will be measured using the Early Treatment Diabetic Retinopathy Study (EDTRS) chart.

For suppression evaluation, the results of the Worth Four-dot Test at 6 m and 33 cm will be interpreted as binocular fusional response, suppression, or diplopia.

Stereoacuity will be examined with Randot Stereotest (Stereo Optical Co, Chicago, Illinois, USA).

Objective refraction will be determined with an auto-refractometer (KR-1Auto Kerato-Refractometer, Topcon, Japan). Cycloplegic refraction will be performed in all patients at baseline and the last follow-up examination.

Compliance with treatment will be calculated by the time recorded in the Excel file in the server. The file contents will provide the user’s activity including days, minutes, and stages played, the frequency of winning and losing, etc.

### Sample size

Based on the result of Kelly et al. study [19], visual acuity outcome and by using the following formula, at the 5% significance level (two-sided) with 80% power, 20 patients would be required per arm. Considering the 10 % dropout rate during the study, our goal is to employ 22 patients in each group (a total number of forty-four participants).

- Group 1: Mean1 ± SD1 = 0.15 ± 0.08
- Group 1: Mean2 ± SD2 = 0.07 ± 0.08

$$ n1=n2=\left[{\left(\mathrm{SD}1\right)}^2+{\left(\mathrm{SD}2\right)}^2{\left({Z}_{1-\alpha /2}+{Z}_{1-\beta}\right)}^2\right]/{\left(\mathrm{Mean}2-\mathrm{Mean}1\right)}^2 $$

### Randomization

The randomization sequence will be created using www.randomization.com. The sequence of the generated random numbers will be transferred to sealed envelopes by an independent researcher (SA), not involved in the data collection or intervention. The corresponding envelopes will be opened only after the target participants signed the informed consent and completed all baseline assessments (JM). Therefore, eligible participants will be randomly divided into two groups to either the patch therapy or binocular game group.

### Blinding

Due to the nature of the intervention, the use of red-green anaglyphic glasses does not allow patients to be blind. However, a certified optometrist as the outcome assessor (MN) will be unaware of patient grouping. Moreover, there was no possible access to the database of the results of patients for the certified examiner. Also, the data manager (who generated the randomization sequence, prepared envelopes and maintained a list of enrolled participants) and the data analyzer will be completely blinded to the control and intervention group characteristics.

### Data monitoring and management

We formed a management committee (MER, SA, MN) to monitor data quality and also approve any decisions regarding this trial. Data entry and coding will be conducted by people other than the research team and checked by the management committee through range checks for data values.

### Statistical analysis

User’s activity in game including days, minutes, and stages played will analyze based on following subgroups:

- Days played (< 30 days, 30 < days < 60, and > 60 days)
- Minutes played (< 500 min, 500 < < 1000, 1000 < < 1500, 1500 < < 2000, > 2000)
- Stages played (< 10, 10 < < 20, > 20)

Also, we collect stereopsis, spherical equivalent in involved and non-involved eyes, and fusional state as the baseline characteristics that have effect on visual acuity. We will use these characteristics in the final analysis.

The normal quantitative variables (based on the Kolmogorov-Smirnov normality test) will be described using mean and standard deviation. The remaining data will be described using the median and interquartile range. To test the mean difference of quantitative variables between the control and intervention groups, if the normality assumption is met, an independent-sample *T* test will be used. Otherwise, Mann-Whitney *U* test will be used to compare the data. To test the homogeneity of the qualitative variables in the two groups, the chi-squared test and Fisher’s exact test will be used at the *p* value of 0.05. Repeated measures ANOVA or non-parametric tests will be applied to continuous outcomes measured repeatedly. All statistical tests will be two-sided at the 5 % significance level. Statistical analyses will be performed using SPSS version 22. The analysis will be performed on an intention to treat and also per protocol approaches. No interim analyses are planned.

## Results

So far, the need analysis, design procedure, and pilot test of intervention are fulfilled. The recruitment of participants has not been completed and is scheduled to end in September 2021. The Pivot game is potentially useful to improve amblyopic-eye visual acuity outcomes.

## Discussion

This RCT builds upon previous trials requiring further evaluation of the effectiveness of binocular game treatment for children with amblyopia, particularly mild to moderate anisometropic cases without a prior treatment except for refractive correction.

The newly designed binocular dichoptic game in this study consists of specific, well-defined characteristics, including the duration of playing the game, no need to attenuate the contrast of fellow eye to the level of the amblyopic eye, and change in difficulty of the game and mini-games. The results will also assess compliance by recording the time spent playing.

If the binocular playing game treatment works, it possibly manages to reduce the psychological pressure on families to patch their children’s amblyopic eye or may promote further compliance to the treatment or possibly associates with better binocular function outcomes. Moreover, playing binocular games allows the assessment of the user’s activity in the shortest time by the physician. If the results are favorable, children’s habits and interest in video games can be possibly used for healthcare purposes.

### Strengths and limitations

- The newly designed binocular dichoptic video game in this study was developed in a structured way with the participation of a multi-disciplinary team
- The home-based game consists of specific, well-defined characteristics, including the duration of playing the game, change in difficulty of the game, and mini-games, which distinguishes it from previous similar games
- The design of this study (randomized controlled trial) tends to meet the highest level of evidence
- We cannot blind our patients due to the nature of the intervention, and it is one of our limitations in this study

## Ethics and dissemination

The Ethics Committee of Mashhad University of Medical sciences’ approval date was February 28, 2018, with a reference code of IR.MUMS.fm.REC.1396.783. This trial is registered in Iran Trial Registrar under the registration number: IRCT20180217038768N and registration date 22 April 2019. The results will be disseminated in a peer-reviewed journal.

The dataset that supports the findings of this study is available from the corresponding author upon reasonable request. Personal information about potential and enrolled participants will be stored on a secure file server research drive at MUMS to ensure confidentiality protection before, during, and after the study.

### Ancillary and post-trial care

In case of any probable adverse event (e.g., diplopia), the study treatment will cease and appropriate care will be provided by the physicians in the research team for compensation probable harm.

### Trial status

The study is currently recruiting and enrolling participants according to version 2 of the protocol in July 2020. Recruitment began on May 31, 2019, and the expected recruitment end date will be the end of September 2021.

## Supplementary Information

**Additional file 1.** SPIRIT 2013 checklist: Recommended items to address in a clinical trial protocol and related documents.

## Data Availability

The datasets used and/or analyzed during the present study are available from the corresponding author (SA) upon reasonable request.

## Abbreviations

ETDRS: Early Treatment for Diabetic Retinopathy Study
LogMAR: Logarithm of the minimum angle of resolution
RCT: Randomized controlled trial
BCVA: Best corrected visual acuity
UCVA: Uncorrected visual acuity
